# Unraveling Palisaded Encapsulated Neuroma: A Case Report and Diagnostic Challenges

**DOI:** 10.7759/cureus.70256

**Published:** 2024-09-26

**Authors:** Kawaiola Cael Aoki, Joseph Gofman, Merrick D Elias

**Affiliations:** 1 Medicine, Nova Southeastern University Dr. Kiran C. Patel College of Osteopathic Medicine, Fort Lauderdale, USA; 2 Dermatology, Nova Southeastern University Dr. Kiran C. Patel College of Osteopathic Medicine, Fort Lauderdale, USA; 3 Dermatology, Elias Dermatology, Pembroke Pines, USA

**Keywords:** dermato-pathology, neuroma, rare skin lesion, skin histopathology, skin lesions

## Abstract

Palisaded encapsulated neuroma (PEN), also known as solitary circumscribed neuroma, is a rare, benign tumor of neuronal origin, often located on the head or neck. We present a case involving a 77-year-old Middle Eastern woman with a slowly enlarging, slightly symptomatic lesion on her upper lip. Histopathological analysis following a shave biopsy revealed the lesion to be a PEN. Accurate diagnosis is crucial to avoid unnecessary testing or treatment for mimicking conditions such as basal cell carcinoma or neurofibroma. Following the removal of the patient's lesion, no additional treatment was necessary, and there was no recurrence.

## Introduction

Palisaded encapsulated neuroma (PEN) is a rare, benign intraneural tumor, usually presenting as an asymptomatic, well-circumscribed, firm, intradermal nodule in middle-aged adults with no gender predilection [1,2]. It often arises on the head and neck, usually on the face near the margin of a mucocutaneous junction, and less commonly on the trunk, shoulder, arm, hand, foot, oral and nasal cavity, and penis [2-4]. The lesion measures 2-6 mm, enlarging gradually over several years, and its color can range from whitish gray to skin-colored or pink [1,2]. No hairs grow from its surface; telangiectasias are minimal or absent, and ulceration may only be present secondary to trauma. Necrosis and hemorrhage have not been observed [2,4].

Clinically, PEN may be confused with other entities, such as melanocytic nevi, basal cell carcinoma (BCC), and other adnexal tumors, due to its non-distinct appearance. This makes accurate diagnosis crucial to avoid unnecessary treatments. Here, we document a case that illustrates how a suspicious facial lesion was later diagnosed as a PEN following a biopsy.

## Case presentation

A 77-year-old Middle Eastern woman presented with a multi-year history of a 3 mm flesh-colored to slightly erythematous papule with central monochromatic hyperpigmentation on the left upper lip. The lesion was gradually enlarging and mildly symptomatic, with no history of trauma or prior treatment. There was no indication or history suggestive of melanoma or neurofibromatosis. The lesion was firm and non-tender on clinical examination, without any palpable regional lymphadenopathy. Palpation revealed that the lesion was fixed to the overlying skin but not to the underlying structures. There were no signs of induration or fluctuance. A dermoscopic examination did not reveal any specific features.

**Figure 1:**
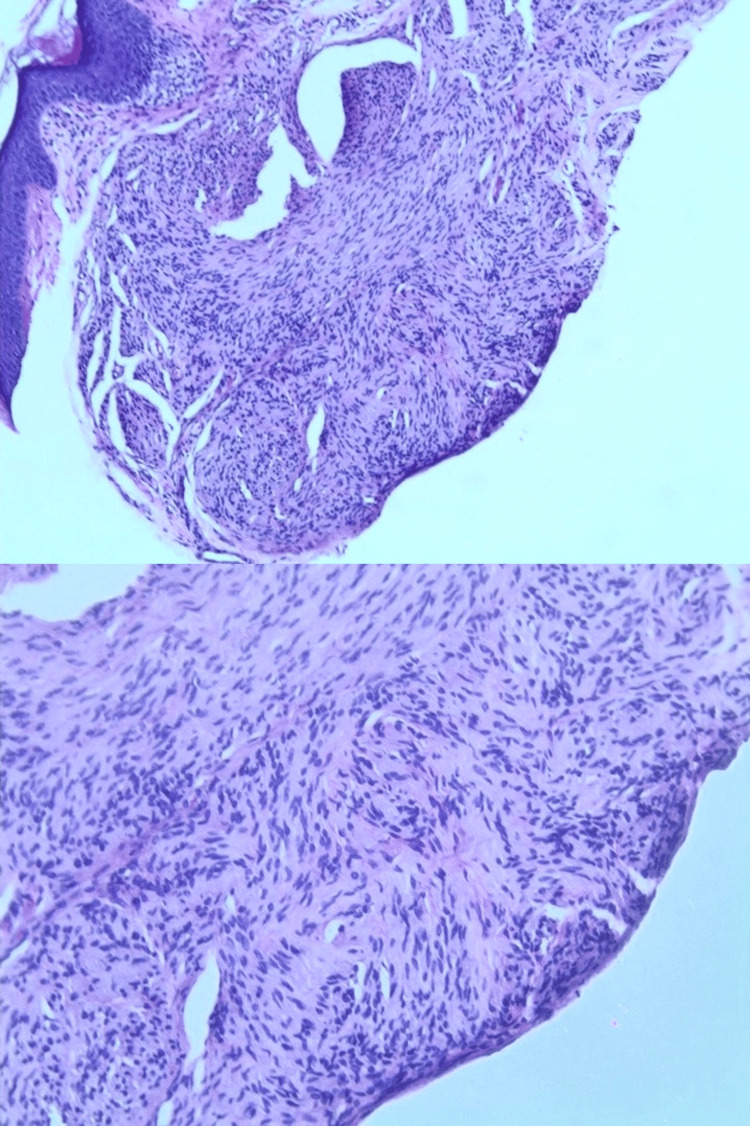
Shave biopsy of upper lip lesion Histopathological image showing encapsulated dermal lobules of interlacing Schwann cells, with variable numbers of fine axons and myelin sheath remnants, without prominent palisading of nuclei (hematoxylin-eosin stain).

A shave biopsy revealed interlacing Schwann cells with fine axons and myelin sheath remnants encapsulated in dermal lobules, without prominent palisading of nuclei (Figure 1). Histological analysis showed that the lesion was well-circumscribed and lacked features suggestive of malignancy. The tumor cells were positive for S100, collagen type IV, and vimentin, but negative for epithelial membrane antigen (EMA). Perineural cells (capsule) were positive for EMA. The lesion was diagnosed as a PEN, and no further treatment was deemed necessary.

## Discussion

PEN consists of the hyperplastic proliferation of typical elements of peripheral nerve fibers [3]. As palisading and encapsulation are not always seen, PEN has been called solitary circumscribed neuroma (SCN). Still, it is not exclusively solitary or circumscribed [1,3]. The clinical appearance may raise concerns about a melanocytic nevus, adnexal neoplasm, or BCC [3]. Unlike BCC, PEN and SCN will have circumscription, sparse telangiectasias, and a long duration of the tumor without ulceration [4].

Histopathologically, PEN and SCN may mimic other peripheral nerve sheath tumors: schwannoma, neurofibroma, traumatic neuroma, and leiomyoma [2,3]. Schwannoma is usually subcutaneous, with Antoni-A and Antoni-B areas, Verocay bodies, a thick collagenous capsule, and absent or sparse axons, compared to the thinner perineurium and abundant axons of PEN and SCN [2,3]. Neurofibroma is rich in mucopolysaccharide, lacks a capsule, and has fewer axons; traumatic neuroma, while histologically similar to PEN and SCN, has inflammatory cells and scarring; and leiomyoma contains smooth muscle cells that stain positive for desmin and vimentin [2]. A detailed differential diagnosis, including clinical presentation, histological features, diagnostic tests, and prognosis for conditions that may mimic PEN, can be found in Table 1 [5].

**Table 1:** Differential diagnosis for palisaded encapsulated neuroma Table credit: [5]

Differential diagnosis	Clinical presentation	Histology	Tests	Prognosis
Amelanotic melanoma	Red or skin-colored nodule, often mistaken for basal cell carcinoma (BCC)	Atypical melanocytes throughout the dermis	Dermoscopy, biopsy, immunohistochemistry (S100, HMB-45)	Aggressive, high risk of metastasis if not diagnosed and treated early
Angioleiomyoma	Painful nodule, often on lower extremities	Similar to leiomyoma but with prominent vascular channels	Biopsy, immunohistochemistry	Benign, can be painful; typically does not recur after excision
Basal cell carcinoma	Pearly papule with telangiectasias, often ulcerates	Basaloid cells with peripheral palisading and clefting	Biopsy with histopathological examination	Good if detected early; can be locally invasive but rarely metastasizes
Epidermal cyst	Subcutaneous nodule with central punctum, may be inflamed	Cyst lined by stratified squamous epithelium with a granular layer and filled with keratinous material	Ultrasound, biopsy	Benign, can become inflamed or infected if ruptured
Fibrous papule	Firm, skin-colored papule, typically on the face	Fibroblasts and collagen bundles in a stroma	Clinical examination, dermoscopy, biopsy	Benign, no malignant potential
Leiomyoma	Firm, painful, skin-colored nodule	Intersecting bundles of smooth muscle cells, positive for desmin and vimentin	Biopsy, immunohistochemistry	Benign, can be painful; typically does not recur after excision
Melanocytic nevus	Well-circumscribed pigmented macule or papule	Nests of melanocytes at the dermo-epidermal junction and/or in the dermis	Dermoscopy, biopsy	Benign; rare transformation to melanoma
Neurofibroma	Soft, flesh-colored papules or nodules, may be multiple	Spindle cells with wavy nuclei in a myxoid stroma with collagen fibers, absence of encapsulation	Biopsy, genetic testing for NF1 if multiple	Benign, but neurofibromas associated with NF1 have a risk of malignant transformation
Schwannoma	Solitary, firm, mobile nodule	Antoni A (cellular) and Antoni B (less cellular) areas, Verocay bodies, lack axons	MRI, biopsy	Benign, excellent prognosis; rarely, malignant transformation
Traumatic neuroma	Painful nodule at the site of previous injury or surgery	Disorganized proliferation of nerve fascicles with fibrosis and inflammatory cells	Clinical history, biopsy	Benign, may require surgical excision for symptomatic relief

The correct diagnosis of PEN and SCN is essential because they are benign, unlike their mimics. Neurofibromas (associated with neurofibromatosis type I) have a predilection for malignancy. Multiple endocrine neoplasia syndrome type 2B (MEN 2B) presents with multiple mucosal neuromas and is associated with occult thyroid medullary carcinoma and pheochromocytoma [4]. Misdiagnosing these lesions may lead to unnecessary worry and testing. The treatment of choice is total excision, which is curative. Even after incomplete excision, PEN and SCN do not recur, suggesting that their origin is reactive rather than neoplastic. No further testing or concern for systemic disease or malignancy is warranted [2].

## Conclusions

PEN is a rare, non-cancerous tumor that is usually found on the head or neck. Accurate diagnosis of PEN is crucial to avoid unnecessary testing and treatment, as it can mimic other, more concerning conditions, such as BCCs or neurofibromas. The presented case of a 77-year-old Middle Eastern woman highlights the significance of recognizing PEN's distinctive clinical and histopathological features, which can be managed effectively through total excision. This underscores the importance of including PEN in the differential diagnosis, ensuring targeted and effective medical management while minimizing patient distress and unwarranted interventions.
